# Analysis of Polycyclic Aromatic Hydrocarbon (PAH) Mixtures Using Diffusion-Ordered NMR Spectroscopy and Adsorption by Powdered Activated Carbon and Biochar

**DOI:** 10.3390/ma11040460

**Published:** 2018-03-21

**Authors:** Dong An, Chengchen Guo, Yanan Chen

**Affiliations:** 1 Department of Environmental Science & Engineering, Fudan University, 220 Handan Road, Shanghai 200433, China; 16210740021@fudan.edu.cn; 2 Shanghai Institute of Pollution Control and Ecological Security, Shanghai 200092, China; 3 Department of Biomedical Engineering, Tufts University, Medford, MA 02155, USA; chengcg@hotmail.com

**Keywords:** PAH, analysis, NMR, adsorption, activated carbon

## Abstract

Analysis of polycyclic aromatic hydrocarbons (PAHs) in air and water sources is a key part of environmental chemistry research, since most PAHs are well known to be associated with negative health impacts on humans. This study explores an approach for analyzing PAH mixtures with advanced nuclear magnetic resonance (NMR) spectroscopic techniques including high-resolution one-dimensional (1D) NMR spectroscopy and diffusion-ordered NMR spectroscopy (DOSY NMR). With this method, different kinds of PAHs can be detected and differentiated from a mixture with high resolution. The adsorption process of PAH mixtures by PAC and biochar was studied to understand the mechanism and assess the method.

## 1. Introduction

Polycyclic aromatic hydrocarbons (PAHs) are a large group of chemicals with 2 to 7 fused aromatic rings. Many PAHs are well known to be associated with environmental pollutions and negative health impacts on humans [1,2,3]. They have been designated as one of the primary suspected carcinogens among atmospheric pollutants, which may increase the health risks, such as cancer and respiratory distress in humans [4,5,6]. To date, the most potent PAH carcinogens that have been identified are benzo[a]pyrene, benzo[a]anthracene, and dibenz[a,h]anthracene. Generally, PAHs are released into the environment from natural and anthropogenic sources [7]. Natural sources include forest fires and volcanic eruptions, while anthropogenic sources primarily include incomplete burning of fuels, traffic exhaust, oil spills, and cooking [8]. Due to a variety of release sources and physicochemical properties that allow for PAHs to be transported over long distances before deposition through atmospheric precipitation, PAHs are widely distributed in the atmosphere, soil, and water. Therefore, the detection and trace analysis of PAHs is of significant importance for reducing the negative health impact of PAHs on humans.

Standard methods, primarily based on gas chromatography (GC), are commonly used to determine the composition of mixtures of organic compounds [9]. However, this requires a suitable column and detectors for variable targets to achieve sensitive and accurate analysis. Additionally, it suffers from signal overlap due to the similar physicochemical properties of the compounds. Recently, NMR spectroscopy has shown great potential in environmental research, since it is able to study liquids, gels, solids, gases, and multiphase samples and produce rapid, reliable, and highly sensitive results [10,11,12]. Quantitative NMR spectroscopy can also provide accurate quantitative information about the sample constituents [13]. Unlike GC, NMR spectroscopy does not require multiple detectors, and it is capable of analyzing mixtures with advanced spectroscopic methods, such as multi-dimensional spectroscopy and diffusion-ordered NMR spectroscopy [10,14,15,16].

Diffusion-ordered NMR spectroscopy (DOSY) is a technique that combines radio-frequency pulses used in routine NMR spectroscopy and magnetic field gradients that encode spatial information [17,18]. With this methodology, DOSY is recognized as a convenient NMR method for the analysis of mixtures [19]. The application of DOSY to determine the composition of mixtures is based on the differences in the diffusion coefficients of the individual components. In DOSY, the results are displayed as a two-dimensional (2D) spectrum in which signals are dispersed according to chemical shift in one dimension and diffusion coefficient in the other. Since different molecules possess unique diffusion coefficients that depend on their molecular sizes and their affinity for the solvent, it is possible to separate them out in 2D DOSY spectrum.

In this work, we perform a model study to show the potential of DOSY NMR in analyzing polycyclic aromatic hydrocarbon mixtures by using multiple common polycyclic aromatic hydrocarbons. Also, the isothermal adsorption of PAHs mixtures was conducted to understand the adsorption process and access the analytical methods.

## 2. Experiments

### 2.1. Chemicals and Materials

Benzene, naphthalene, anthracene, phenanthrene, fluoranthene, 1,2-benzanthracene, benzo[a]pyrene, and benzo[e]pyrene were purchased from Sigma Aldrich (St. Louis, MO, USA). Deuterated chloroform (CDCl_3_) was purchased from Cambridge Isotope Inc (Tewkesbury, MA, USA). All of the chemicals were used as received. To prepare single-solute NMR samples, approximately 2.0 mmol of each compound was dissolved in 800 μL CDCl_3_. Three mixture samples were prepared in this work, named Mixture A, Mixture B, and Mixture C. For the preparation of Mixture A, 2.0 mmol each of benzene, naphthalene, anthracene, and phenanthrene were mixed and dissolved in 800 μL CDCl_3_. For the preparation of Mixture B, 2.0 mmol each of benzo[a]pyrene and benzo[e]pyrene were mixed and dissolved in 800 μL CDCl_3_. For the preparation of Mixture C, 2.0 mmol each of benzene, naphthalene, anthracene, phenanthrene, fluoranthene, 1,2-benzanthracene, benzo[a]pyrene, and benzo[e]pyrene were mixed and dissolved in 800 μL CDCl_3_. Different concentrations of benzene, naphthalene, anthracene, and phenanthrene were also prepared to perform adsorption kinetics tests by different kinds of powdered activated carbon (PAC) and biochar. Wood-based powdered activated carbon (200 mesh) was purchased from Calgon Carbon Corp. (Pittsburgh, PA, USA). Biochar was derived from the waste of straw carbonization. Virgin materials characterized by the following parameters, as shown in Table 1. All of the experiments were conducted at room temperature. Mixture A was used to perform as target compounds. The adsorption experiments were conducted in a batch test. The adsorption time was 2 h to guarantee an adsorption balance.

### 2.2. NMR Spectroscopy

Nuclear magnetic resonance (NMR) analysis was conducted on a Varian 500 spectrometer (CA, USA), equipped with ^1^H/^13^C/^15^N 5 mm XYZ PFG triple-resonance probe using standard VNMRJ software (Varian Inc., Palo Alto, CA, USA). All of the experiments were performed with a ^1^H 90° pulse of 7.30 μs at 298.15 K. One-dimensional (1D) ^1^H spectra were collected with a sweep width of 8012.8 Hz, an acquisition time of 5.0 s, a recycle delay of 10 s, and eight scans. Diffusion-ordered NMR spectroscopy (DOSY) measurements were performed with DOSY bipolar pulse pair stimulated echo (dbppse) pulse sequence. A pulsed gradient duration (*δ*) of 1.0 ms incremented from 2.5 to 57.0 G cm^−1^ in 15 steps and a pulsed gradient separation (Δ) of 75 ms were used in the measurements. The spectra were collected with a sweep width of 8012.8 Hz, an acquisition time of 5.0 s, a recycle delay of 10 s, and eight scans. The reported spectra were processed using VNMRJ software, and the diffusion coefficients were calculated using Matlab.

## 3. Results and Discussion

### 3.1. ^1^H NMR Spectra of Polycyclic Aromatic Hydrocarbons

**Figure 1:**
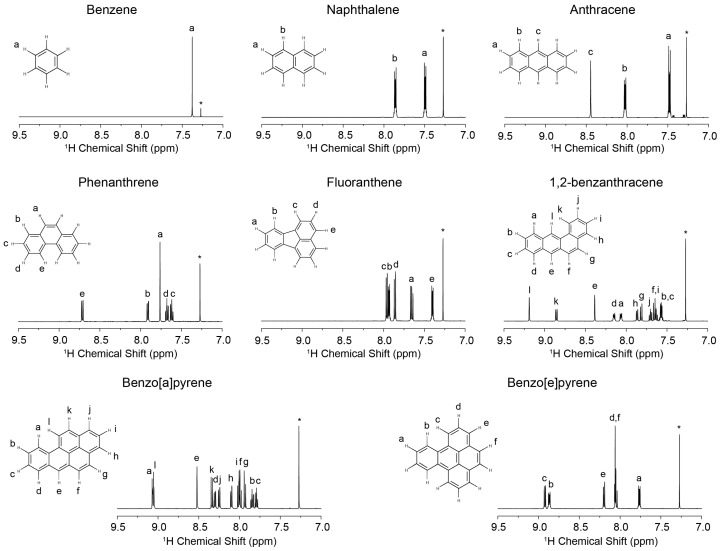
^1^H nuclear magnetic resonance (NMR) spectra and peak assignments in Deuterated chloroform (CDCl_3_) of benzene, fluoranthene, 1,2-benzanthracene, benzo[e]pyrene, benzo[a]pyrene, anthracene, naphthalene, and phenanthrene. * represent CDCl_3_ peak.

**Figure 2:**
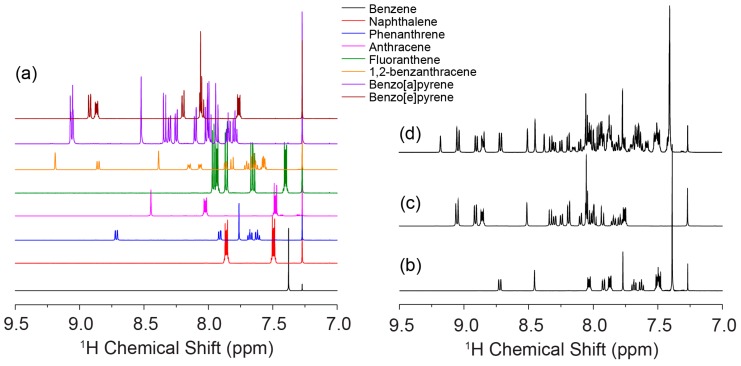
(**a**) Stack plot of ^1^H NMR spectra of benzene, naphthalene, anthracene, phenanthrene, fluoranthene, 1,2-benzanthracene, benzo[a]pyrene, and benzo[e]pyrene; (**b**) ^1^H NMR spectrum of Mixture A; (**c**) ^1^H NMR spectrum of Mixture B; and (**d**) ^1^H NMR spectrum of Mixture C.

^1^H NMR spectra of individual PAHs were collected first to observe the differences among them (Figure 1). For all PAHs, the ^1^H resonances appear in the chemical shift range from 7.0 ppm to 9.5 ppm [20,21]. Based on the stack plot of ^1^H NMR spectra (Figure 2a), most of the PAHs clearly have characteristic peaks that can be used in a DOSY experiment for diffusion coefficient measurement. The ^1^H spectra of three mixtures are presented in Figure 2b–d. For mixture A and mixture B, each species has its own characteristic resonance. However, for mixture C, the resonances from benzene, naphthalene, and fluoranthene show some overlap, which makes it difficult to separate them by DOSY.

### 3.2. Measuring Diffusion Coefficients of Polycyclic Aromatic Hydrocarbons Using DOSY

**Figure 3:**
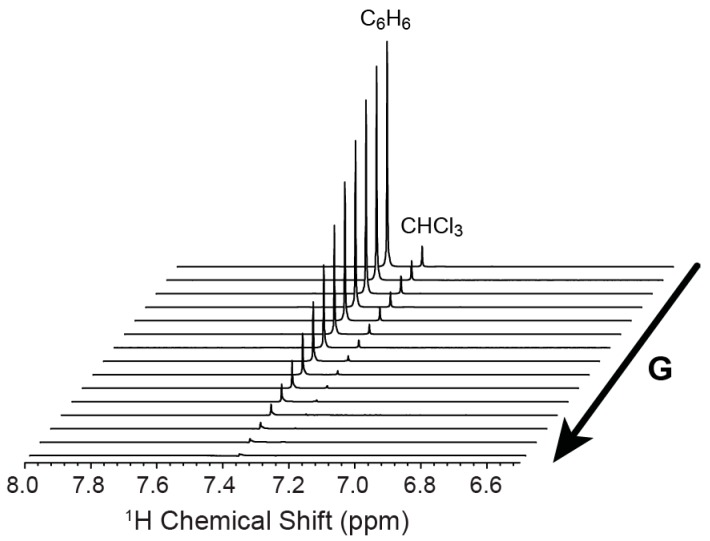
Stack plot of ^1^H NMR spectra of benzene at with increasing gradient field strength (G).

DOSY NMR is a powerful tool to determine the diffusion coefficient of molecules in solvents. Figure 3 shows a typical DOSY 1D spectrum with a variable gradient field. By increasing the gradient field strength, the intensity of the benzene ^1^H resonance decreases dramatically. The observed signal can be described by the Stejskal-Tanner equation [22]:

$$\ln(S(2\tau )/S_{0})=-Dg^{2}\gamma^{2}\delta^{2}(\Delta -\frac{\delta }{3})$$

where *S*(2*τ*) is the signal observed in the experiment, *S*_0_ is the signal observed in the absence of any gradient strength, *g* is the gradient strength, *γ* is the gyromagnetic ratio for the observed nucleus (^1^H in the present study), *δ* is the gradient length, ∆ is the diffusion delay, and *D* is the diffusion coefficient. By plotting the ln(*S*(2*τ*)/*S*_0_) vs. *g*^2^*γ*^2^*δ*^2^(∆ − *δ*/3) (Stejskal-Tanner plot), the diffusion coefficient can be determined from the slope of the curve.

**Figure 4:**
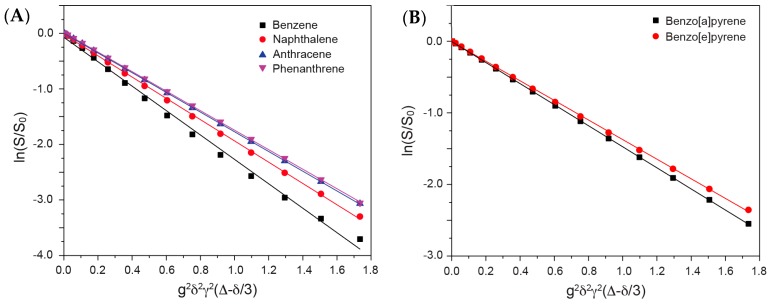
Stejskal-Tanner plot for each compound in Mixture A (**A**) and Mixture B (**B**).

**Figure 5:**
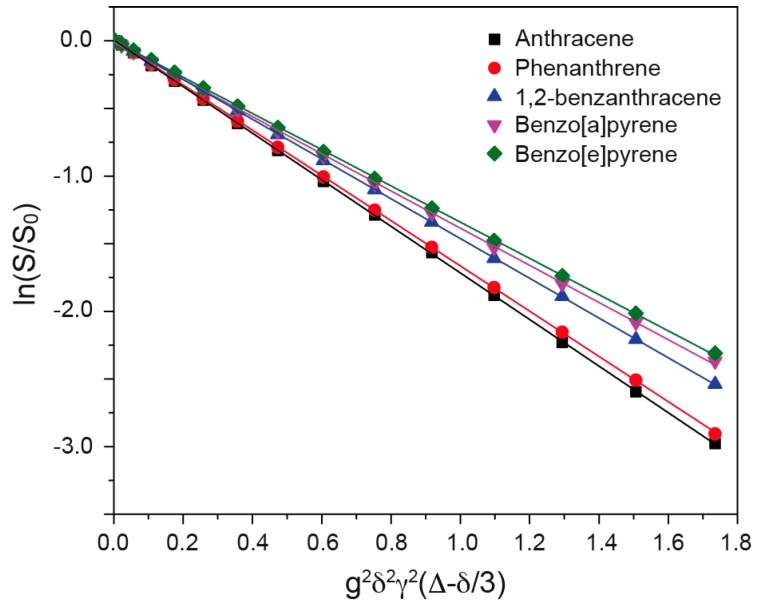
Stejskal-Tanner plot for each species in Mixture C.

In the present study, DOSY NMR was applied to determine the diffusion coefficients of PAHs in the mixtures. By analyzing the characteristic regions for individual PAHs, the diffusion coefficients for well-separated PAHs can be obtained in one single DOSY spectrum. Figure 4 shows the Stejskal-Tanner plots for mixture A and mixture B. All of the compounds in the mixtures were well detected, and the calculated corresponding diffusion coefficients are summarized in Table 2. For mixture C, the Stejskal-Tanner plot is shown in Figure 5. Only five compounds (anthracene, phenanthrene, 1,2-benzanthracene, benzo[a]pyrene, and benzo[e]pyrene) can be clearly separated and detected due to the severe overlap between benzene, naphthalene, and fluoranthene. Additionally, the diffusion coefficients determined from mixture C for the compounds are slightly different from those that are determined from mixture A and mixture B, due to the small variations in the solution compositions.

### 3.3. Analysis of PAH Mixtures Using NMR Spectroscopy

**Figure 6:**
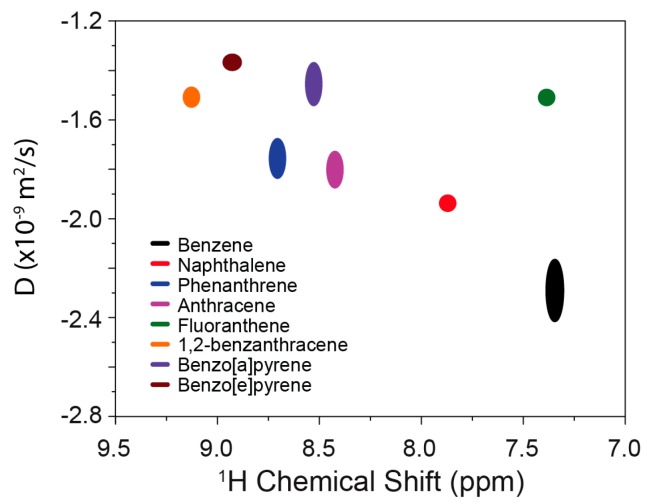
Diffusion-ordered NMR spectroscopy (DOSY) plot for different polycyclic aromatic hydrocarbons in CDCl_3_.

NMR offers a good way to analyze PAH mixtures. Combining high-resolution 1D NMR spectroscopy and DOSY NMR spectroscopy enables the detection and analysis of different components in the mixture. Due to the variable molecular size and diffusion behavior in the solution, different PAHs possess characteristic diffusion coefficients. With DOSY spectroscopy, these PAHs can be well separated and detected in the second dimension (diffusion coefficient dimension). As shown in Figure 6, multiple PAHs can be detected from a mixture by combining chemical shift analysis and diffusion coefficient analysis. This method provides a reliable and cost-efficient way to analyze the PAH mixtures. Moreover, when compared to GC, NMR spectroscopy does not require individual detectors, and it is a non-invasive analytical method.

### 3.4. Isothermal Adsorption

**Figure 7:**
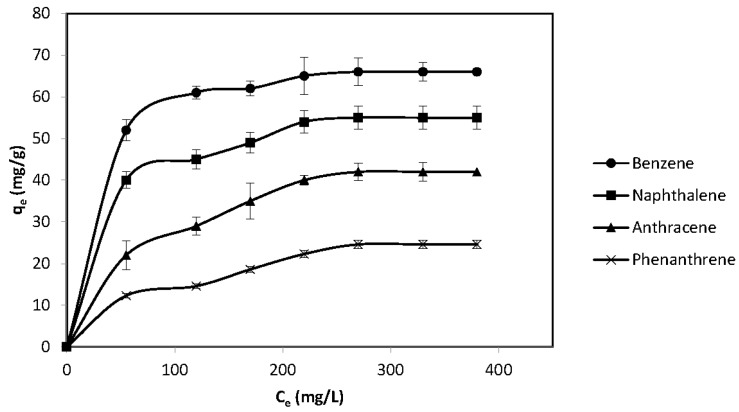
The isothermal adsorption of benzene, naphthalene, anthracene, and phenanthrene by wood-based powdered activated carbon (PAC) (pH = 7 at room temperature).

**Figure 8:**
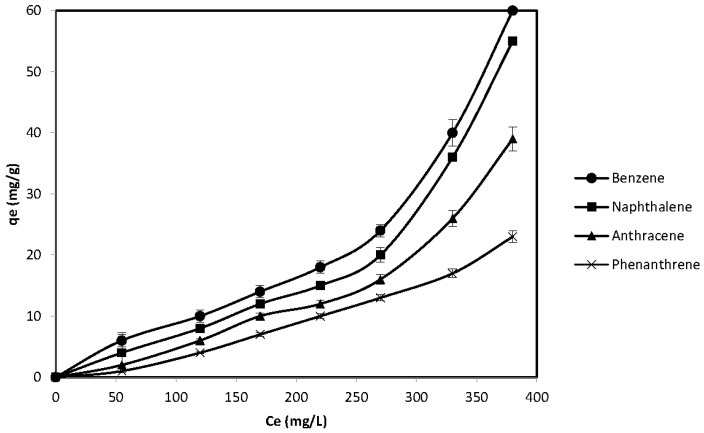
The isothermal adsorption of benzene, naphthalene, anthracene, and phenanthrene by biochar (pH = 7 at room temperature).

The isothermal adsorption of PAH mixtures, including benzene, naphthalene, anthracene, and phenanthrene by wood-based PAC and biochar was conducted to understand the adsorption process and access the analytical methods. As shown in Figure 7 and Figure 8, in the PAH mixtures the highest adsorption removal by PAC and biochar was 66.3 and 60.1 mg/g for Benzene, respectively. Along with the increasing of molecular weights for PAH mixtures, the removal rate decreased significantly. The results showed a lowest removal rate of 23.4 mg/g and 20 mg/g for phenanthrene by PAC and biochar, which has the biggest molecular weight of 228 among four kinds of PAH mixtures. The experimental results about the adsorption capability by PAC and biochar are consistent with previous studies. Previous study [23] found that the samples from industrial drainage received an adsorption capability of 54.8–72.4 mg/g by different PACs. The mechanism of PAHs adsorption by PAC was assumed to be monolayer adsorption that after saturation the adsorption capability was no longer increased, as can be seen in Figure 7. However, the adsorption by biochar showed an increasing process when Ce increased to more than 380 mg/L in Figure 8. The inner structure and charge distribution of biochar allows for the occurrence of multi-layer adsorption. The mechanism will be explored by X-ray photoelectron spectroscopy (XPS) and Fourier transform infrared spectroscopy (FTIR) in our future research.

## 4. Conclusions

This study demonstrates a model analyzing PAH mixtures by NMR spectroscopy. Combining high-resolution 1D NMR spectroscopy and DOSY NMR spectroscopy allows for the detection and differentiation of different kinds of PAHs in the mixture with high resolution. This reliable, convenient, and cost-effective method has great potential to serve as a powerful tool for environmental sample analysis. The novel method was successfully applied to the determination of PAH mixtures in adsorption processes by PAC and biochar in batch tests.

## Figures and Tables

**Table 1:** Characteristics parameters of virgin materials for adsorption tests.

Adsorbent	Iodine Number (mg/g)	Methylene Number (mg/g)	BET Sufface Area (m^2^/g)
Wood-based PAC	910	120	1100
Biochar	1400	95	880

**Table 2:** Summary of diffusion coefficients of compounds in different mixture solutions.

Solutes	Integration Region (ppm)	Single (m^2^/s) × 10^9^	Mixture A (m^2^/s) × 10^9^	Mixture B (m^2^/s) × 10^9^	Mixture C (m^2^/s) × 10^9^
Benzene	7.34~7.40	2.43 ± 0.02	2.20 ± 0.04	-	-
Naphthalene	7.81~7.88	1.93 ± 0.01	1.92 ± 0.01	-	-
Phenanthrene	8.67~8.75	1.83 ± 0.01	1.76 ± 0.01	-	1.67 ± 0.01
Anthracene	8.41~8.47	1.86 ± 0.01	1.77 ± 0.01	-	1.72 ± 0.01
Fluoranthene	7.35~7.43	1.53 ± 0.01	-	-	-
1,2-benzanthracene	9.14~9.22	1.55 ± 0.01	-	-	1.47 ± 0.01
Benzo[a]pyrene	8.49~8.54	1.55 ± 0.01	-	1.47 ± 0.01	1.37 ± 0.01
Benzo[e]pyrene	8.88~8.95	1.40 ± 0.01	-	1.37 ± 0.01	1.34 ± 0.01
